# Piloting a generic cancer consumer quality index in six European countries

**DOI:** 10.1186/s12885-016-2752-9

**Published:** 2016-09-02

**Authors:** Anke Wind, Mark Patrick Roeling, Jana Heerink, Herman Sixma, Pietro Presti, Claudio Lombardo, Wim van Harten

**Affiliations:** 1 Division of Psychosocial Research and Epidemiology, The Netherlands Cancer Institute-Antoni van Leeuwenhoek Hospital, Plesmanlaan 121, 1066 CX Amsterdam, The Netherlands; 2 Department of Health Technology and Services Research, University of Twente, P.O. Box 217, 7500 AE Enschede, The Netherlands; 3 Department of Computer Science, University of Oxford, Parks Road, OX1 3QD Oxford, UK; 4 Netherlands Institute for Health Services Research (NIVEL), P.O. box 1568, 3500 BN Utrecht, The Netherlands; 5 European Cancer Patient Coalition (ECPC), 40 rue de Montoyer, Brussels, Belgium; 6 Department of Management, University of Torino, C.so Unione Sovietica 218 Bis, 10134 Turin, Italy; 7 Organisation of the European Cancer Institute’s (OECI), 11 Rue d’Egmont B-1000, Brussels, Belgium; 8 SOS Europe Srl, Via delle Campanule, 74, 16148 Genova, Italy; 9 CEO Rijnstate Hospital, Arnhem, The Netherlands

**Keywords:** Consumer Quality Index (CQI), Healthcare evaluation, Healthcare quality, Patient experience, Patient satisfaction

## Abstract

**Background:**

Accounting for patients’ perspective has become increasingly important. Based on the Consumer Quality Index method (founded on Consumer Assessment of Healthcare Providers and Systems) a questionnaire was recently developed for Dutch cancer patients. As a next step, this study aimed to adapt and pilot this questionnaire for international comparison of cancer patients experience and satisfaction with care in six European countries.

**Method:**

The Consumer Quality Index was translated into the local language at the participating pilot sites using cross-translation. A minimum of 100 patients per site were surveyed through convenience sampling. Data from seven pilot sites in six countries was collected through an online and paper-based survey. Internal consistency was tested by calculating Cronbach’s alpha and validity by means of cognitive interviews. Demographic factors were compared as possible influencing factors.

**Results:**

A total of 698 patients from six European countries filled the questionnaire. Cronbach’s alpha was good or satisfactory in 8 out of 10 categories. Patient satisfaction significantly differed between the countries. We observed no difference in patient satisfaction for age, gender, education, and tumor type, but satisfaction was significantly higher in patients with a higher level of activation.

**Conclusion:**

This European Cancer Consumer Quality Index(ECCQI) showed promising scores on internal consistency (reliability) and a good internal validity. The ECCQI is to our knowledge the first to measure and compare experiences and satisfaction of cancer patients on an international level, it may enable healthcare providers to improve the quality of cancer care.

**Electronic supplementary material:**

The online version of this article (doi:10.1186/s12885-016-2752-9) contains supplementary material, which is available to authorized users.

## Background

The organization of care for cancer patients is complex and multifaceted, cancer can cause a great deal of distress for patients. A study among lung-cancer patients showed that 27 % mentioned healthcare experiences as an important cause of distress. Waiting times, and lack of information are some mentioned experiences [1]. Different healthcare providers are engaged in prevention, diagnosis, treatment and follow up. This requires a high degree of coordination and if inadequately organized, can result in fragmented and discontinued care [2]. The Institute of Medicine (IOM) proposed patient centeredness as a way how healthcare systems could improve patients’ experience [3]. Patient centeredness is defined as: care that respects and responds to individual patient’s preferences, needs, and values and involves clinical decisions guided by patients [3]; and is associated with better treatment adherence and improved health outcomes [4]. Healthcare professionals and patients do not always agree on what is important in patient centered care. Wessels et al. [5] reported that expertise and attitude of healthcare providers as well as accessibility were more important to cancer patients than healthcare professionals expected. This underlines the importance of questionnaires that actually reflect the perspective of the patient. Patient experience and satisfaction are increasingly seen as a quality outcome for health-system or –provider performance, by consumers, practitioners and governing agencies [6].

The Consumer Quality Index (CQI) used in this study is based on the American CAHPS (Consumer Assessment of Healthcare Providers and Systems) [7]. The CAHPS is one of the most well-known initiatives to measure quality of care from the healthcare user’s perspective. CAHPS is widely used in the United States and translated and used in the Netherlands. The CQI is also based on the Dutch QUOTE (Quality of care through the patient’s eyes) [8]. Many researchers have designed instruments to measure patient experience and satisfaction that are specific to a country’s health system or individual hospital [9–14]. In order to compare performance across health systems and providers, standardized and comparable measures of patient experience and satisfaction are necessary, to our knowledge there is no such instrument yet. Our objective was to adapt and test the psychometric properties of a generic questionnaire that measures the actual experiences and satisfaction of cancer patients with care in different countries in Europe based on the Dutch version of the CQI. A generic questionnaire has advantages: it can be used for patients with all tumor types, which makes developing different tumor-specific questionnaires redundant [4]. Questions regarding actual experiences tend to reflect the quality of care better and are more interpretable and actionable for quality improvement purposes, while satisfaction ratings shows whether expectations were met [15]. In order to get a comprehensive picture both satisfaction and experience are measured. Our research questions were:

1. *What are the differences in patient experience and satisfaction between countries and/or patient characteristics?*
2. *What is the validity and internal consistency (reliability) of the European Cancer Consumer Quality Index?*

## Methods

### Questionnaire

To use the existing CQI in an international context, questions related specific to the Dutch system were removed based on expert opinion. The updated questionnaire was send to the European Cancer Patient Coalition and a patient representative at each of the pilot sites to check for appropriateness for international measurement. Patient representatives were asked to judge whether their patients would be able to read and comprehend the questions. Twelve institutes across Europe were invited to participate of which seven institutes in six countries (two in Italy) responded positively. These countries were: Hungary (HUN), Portugal (PRT), the Netherlands (NLD), Romania (ROM), Lithuania (LIT), and Italy (ITA). The CQI was translated into the local language at the pilot sites and translated back into English, to ensure that no information was lost in translation, so called cross-translation. Cross-translation is used to ensure the translated instruments are conceptually equivalent in each of the target countries/cultures [16]. The CQI used in this study will be referred to as European Cancer Consumer Quality Index (ECCQI) and consists of 65 questions/items divided into 13 categories. The three categories with demographic or disease specific information were used as background and were not part of the analysis which therefore includes 10 categories (45 items). Participants were given the opportunity to comment on the questionnaire.

### Data collection

The target response was a minimum of 100 respondents per institute.

Every institute assigned a person who ensured the distribution and collection of the questionnaires. In the Netherlands, data were collected through an online survey tool [17], in other institutes (N = 6) the questionnaire was paper-based due to the fact that internet coverage was not sufficient in these countries. Respondents were selected by convenience sampling. This study was performed in agreement with the declaration of Helsinki. Approval by a medical ethics committee was not required. All participants consented to the use of the data provided by them. Data from interviews and questionnaires were analyzed anonymously.

### Inclusion criteria

The following criteria were used for inclusion of the questionnaires: (1) Patients had to be 18 years or older, (2) patients had to be examined, treated or had after-care for cancer within the last two years in the examined center, (3) gender, age and level of education had to be known, (4) 50 % of the questions answered.

### Cognitive interviews

Cognitive interviews were performed in order to measure the face validity of the ECCQI and to identify problems in the wording or structure of questions which might lead to difficulties in question administration, miscommunication, etc. Face validity is the extent to which a test is subjectively viewed as covering the concept it is supposed to measure, which in this study, is the experience of and satisfaction of cancer patients with care received at the cancer center. Both ‘thinking aloud’ and ‘verbal probing’ [18], were used in this study. When thinking aloud, respondents are asked to read the questions out loud and to verbalize their thoughts as they fill out the questionnaire. With verbal probing, the interviewer asks follow-up questions to understand a participant’s interpretation more clearly and precisely. The cognitive interviews were conducted in the Netherlands, Romania (with interpreter), and Portugal (with interpreter). Data collected through the cognitive interviews were analyzed by means of the Question Appraisal System(QAS-99) [19]. The QAS-99 consists of seven elements: (i) Determine if it is difficult to read the question uniformly for all respondents; (ii) Look for problems with any introductions, instructions, or explanations from the respondent’s point of view; (iii) Identify problems related to communicating the intent or meaning of the question to the respondent; (iv) Determine if there are problems with assumptions made or the underlying logic the questions; (v) Check whether respondents are likely to not know or have trouble remembering information; (vi) Assess questions for sensitive nature or wording, and for bias; (vii) Assess the adequacy of the range of responses to be recorded.

### Recoding

Data were recorded in order to be analyzed. Almost all categories of the CQI consist of questions with four response options: never = 1, sometimes = 2, usually = 3 and always = 4. For the categories that did not consist of those four response options, the options were recorded into one of the four options above. Response codes of the questions about demographic characteristics were also recoded; (i) Age: 18–34, 35–64, and 65 or older; (ii) Years of education: low (1–8 years), moderate (9–13 years), and high (14 and higher). The answers ‘I don’t know/I no longer remember’ and ‘Not applicable’ were scored as missing.

### Analyses

For descriptive analyses we used SPSS v.22. To aid future comparison of samples and normalization, descriptive statistics involved calculating the weighted mean for each scale and country. In line with the instructions [20], patient’s scores were only valid if 50 % or more questions within a scale were answered. We performed a chi-square test to determine whether the distribution of patient characteristics such as age differed between countries. For every category the weighted mean was calculated per country, where the weight depended on the number of items rated by the patient. We summed the scale scores and calculated the weighted mean of overall patient experience and satisfaction for every patient. The possible effects of demographic characteristics on ECCQI score were examined with one way ANalyses Of VAriance (ANOVA) analysis (95 % Confidence Intervals: CI).

To estimate the internal consistency (reliability) of each scale, we calculated the Cronbach’s alpha [21] (Cronbach, 1951; α) for ordinal items. In short, we followed the method from Gadermann et al. [22], where α was calculated on the polychoric correlation matrix (calculated with the *psych* package available in the R programming language), instead of the normal Pearson correlation. Acceptable α scores fall between 0.5 to 0.7 and α is considered good if higher than 0.7 [23].

The ECCQI presented here is based on the factor structure of the CQI. We tested the structural validity of the ECCQI in our data with Confirmatory Factor Analysis (CFA). The rationale behind applying CFA is that a predefined measurement model can be tested with Structural Equation Modeling, where CFA provides insight into the fit of the model on the current data. CFA analyses were conducted in Mplus v.7 [24] fitted using the Weighted Least Squares Mean Variance adjusted (WLSMV). As general measures of fit, the Root Mean Square Error of Approximation (RMSEA) and Comparative Fit Index (CFI) were evaluated. The RMSEA provides an indication of how well the model fits in the population. Values > .10 indicate poor model fit, values between .08 and .05 indicate adequate model fit, and values of .05 or below indicate good fit of the model to the data [25]. The CFI ranges from zero to one and higher values indicate better fit. It has been shown to be an adequate fit statistic for ordinal data [26] with values larger than .90 indicating moderate fit and .95 indicating good fit. Also, we fitted all models using the Weighted Least Squares Mean Variance adjusted (WLSMV).

### Patient activation

To investigate relationships between level of patient activation and ECCQI score the Patient Activation Measure (PAM) was administered [27, 28]. The PAM was included later on in the study. It was only send to institutes in the Netherlands, Romania and Italy, since these countries indicated that they still could implement the PAM at that time, however not all patients filled out the PAM.

## Results

### Response

**Fig. 1:**
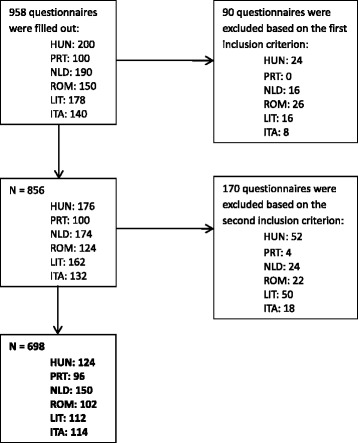
Flow-chart of sample size ECCQI

Initially 958 questionnaires were collected. After application of the inclusion criteria 698 questionnaires were included in this study (see Fig. 1). Respondent characteristics can be found in Table 1. In order to ensure anonymity data are presented by country and not by individual institute (the Italian institutes are combined).

**Table 1:** ECCQI Respondent characteristics. Percentage and absolute numbers

	HUN	PRT	NLD	ROM	LIT	ITA	Total
Age, % (#)							
18–34	3,2 (4)	3,1 (3)	5,3 (8)	5,9 (6)	3,6 (4)	7,0 (8)	4,7 (33)
35–64	57,3 (71)	59,4 (57)	68,7 (103)	78,4 (80)	74,1 (83)	71,1 (81)	68,1 (475)
> 65	39,5 (49)	37,5 (36)	26,0 (39)	15,7 (16)	22,3 (25)	21,9 (25)	27,2 (190)
Gender, % (#)							
Male	22,6 (28)	39,6 (38)	44,0 (66)	27,5 (28)	34,8 (39)	29,8 (34)	33,4 (233)
Female	77,4 (96)	60,4 (58)	56,0 (84)	72,5 (74)	65,2 (73)	70,2 (80)	66,6 (465)
Education, % (#)							
Low	7,3 (9)	56,3 (54)	1,3 (2)	7,8 (8)	13,4 (15)	23,0 (26)	16,4 (114)
Moderate	41,9 (52)	29,2 (28)	24,7 (37)	43,1 (44)	29,5 (33)	54,0 (61)	36,6 (255)
High	50,8 (63)	14,6 (14)	74,0 (111)	49,0 (50)	57,1 (64)	23,0 (26)	47,1 (328)
Activation, % (#)							
Level 1			32,4 (11)	8,6 (5)		27,7 (18)	18,6 (34)
Level 2			47,1 (16)	12,1 (7)		16,9 (11)	18,6 (34)
Level 3			8,8 (3)	53,4 (31)		40,0 (26)	43,7 (80)
Level 4			11,8 (4)	25,9 (15)		15,4 (10)	19,1 (35)
Experienced health % (#)							
Excellent	5,6 (7)	1,0 (1)	4,3 (6)	0	1,8 (2)	5,3 (6)	3,2 (22)
Very good	15,3 (19)	1,0 (1)	17,0 (24)	10,9 (11)	3,6 (4)	13,3 (15)	10,8 (74)
Good	36,3 (45)	37,5 (36)	55,3 (78)	42,6 (43)	42,8 (48)	44,3 (50)	43,7 (300)
Moderate	30,6 (38)	48 (46)	21,3 (30)	36,6 (37)	50,0 (56)	30,1 (34)	35,1 (241)
Poor	12,2 (15)	12,5 (12)	2,1 (3)	9,9 (10)	1,8 (2)	7,0 (8)	7,2 (50)
Type of cancer % (#)							
Digestive organs	6,4 (8)	27,1 (26)	8,7 (13)	16,7 (17)	24,1 (27)	12,3 (14)	15,0 (105)
Lung	4,8 6)	4,2 (4)	7,3 (11)	6,9 (7)	6,2 (7)	5,3 (6)	5,9 (41)
Breast	40,3 (50)	29,2 (28)	32,0 (48)	39,2 (40)	16,1 (18)	43,9 (50)	33,5 (234)
Male reproductive organs	6,4 (8)	8,3 (8)	13,3 (20)	6,9 (7)	10,7 (12)	2,6 (3)	8,3 (58)
Skin	9,7 (12)	1,0 (1)	8,0 (12)	1,0 (1)	0,9 (1)	2,6 (3)	4,3 (30)
Blood, bone marrow and lymph nodes	4,0 (5)	2,1 (2)	2,0 (3)	1,0 (1)	0	9,6 (11)	3,1 (22)
Urinary tract	3,2 (4)	1,0 (1)	4,0 (6)	1,0 (1)	7,1 (8)	0.9 (1)	3,0 (21)
Female reproductive organs	8,9 (11)	6,2 (6)	2,7 (4)	19,6 (20)	23,2 (26)	3,5 (4)	10,2 (71)
Head and neck area	3,2 (4)	4,2 (4)	1,3 (2)	1,0 (1)	2,7 (3)	3,5 (4)	2,6 (18)
Central nervous system	1,6 (2)	0	1,3 (2)	1,0 (1)	0	0	0,7 (5)
Bone or soft tissue	1,6 (2)	1,0 (1)	2,7 (4)	1,0 (1)	0,9 (1)	2,6 (3)	1,7 (12)
Endocrine glands	4,8 (6)	0	0,7 (1)	1,0 (1)	0	0	1,1 (8)
Eye or eye socket	0,8 (1)	0	0	0	0	0	0,1 (1)
Other	0,8 (1)	1,0 (1)	5,3 (8)	2,0 (2)	0,9 (1)	0	1,9 (13)
Multiple forms	3,2 (4)	14,6 (14)	10,7 (16)	2,0 (2)	7,1 (8)	13,1 (15)	8,4 (59)
Years of cancer % (#)							
< 1	14.6 (18)	6,5 (6)	2,0 (3)	28,9 (28)	27,9 (29)	27,4 (31)	16,9 (115)
1–2	58,5 (72)	54,3 (50)	86,7 (130)	54,6 (53)	62,5 (65)	26,5 (30)	58,9 (400)
3–5	13,8 (17)	16,3 (15)	10,0 (15)	12,4 (12)	4,8 (5)	21,2 (24)	13,0 (88)
6–10	8,1 (10)	13.0 (12)	0	4,1 (4)	4,8 (5)	13,3 (15)	6,8 (46)
> 10	4.9 (6)	9,8 (9)	1,3 (2)	0	0	11,5 (13)	4,4 (30)
Treatment received %^a^ (#) (more than 1 answer possible)							
Examinations	68.8 (84)	69,8 (67)	93,6 (132)	59,4 (60)	73,2 (82)	91,1 (103)	76,9 (528)
Operation	56.6 (69)	53,6 (37)	70,2 (99)	44,5 (45)	67,0 (75)	33,6 (38)	52,8 (363)
Radiotherapy	33,6 (41)	28,1 (27)	50,3 (71)	38,6 (39)	14,3 (16)	16,8 (19)	31,0 (213)
Chemotherapy	39,3 (48)	80,2 (77)	39,7 (56)	75,2 (76)	36,6 (41)	68,1 (77)	54,6 (375)
Hormone therapy	9,8 (12)	12,5 (12)	19,1 (27)	8,9 (9)	0,9 (1)	12,4 (14)	10,0 (75)
Immunotherapy	1,6 (2)	8,33 (8)	6,4 (9)	2,0 (2)	1,8 (2)	8,0 (9)	4,7 (32)
Aftercare	15,6 (19)	3,13 (3)	75,9 (107)	1,0 (1)	8,0 (9)	2,7 (3)	20,7 (142)
Stage of treatment % (#)							
Tests to ascertain diagnosis	2,5 (3)	0	0,7 (1)	2,0 (2)	6,4 (7)	0	1,9 (13)
Diagnosis known, will be treated soon	6,7 (8)	2,1 (2)	0,7 (1)	4,0 (4)	9,1 (10)	2,7 (3)	4,1 (28)
Treatment that is intended to cure	37,0 (44)	60,0 (57)	14,7 (21)	66,7 (68)	59,1 (65)	68,7 (77)	48,8 (332)
No further treatment possible	0,8 (1)	0	0	3,9 (4)	0	0	0,7 (5)
Non-curative treatment	5,0 (6)	32,6 (31)	11,2 (16)	10,8 (11)	10,9 (12)	10,7 (12)	12,9 (88)
Check-ups or treatments of the symptoms	39,5 (47)	5,3 (5)	68,5 (98)	11,8 (12)	13,6 (15)	16,1 (18)	28,6 (195)
Finished with treatments and check-ups	8,4 (10)	0	4,2 (6)	1,0 (1)	0,9 (1)	1,8 (2)	2,9 (20)

^a^ percentages indicates percentage of total patients that received that type of treatment

Results of the chi-square test showed a significant difference in the distribution of the patient characteristics such as level of education (*χ*2(10) = 210.315, p < 0.001) and perceived overall health (*χ*2(20) = 77.641, p < 0.001).

### Results of the ECCQI per country

Table 2 shows the descriptive statistics of the ECCQI. The weighted mean of the summed scale scores was 3.35, ranging from 2.05 to 4, being slightly skewed (skewness = .871). Comparison between countries revealed a significant difference in experience and satisfaction [F(5692) = 5.337, p < 0.001]. Post hoc comparisons indicated that this overall effect was predominantly influenced by a significant (p < 0.001) mean difference between Hungary (mean = 3.29, Standard Deviation (StDev) = .34) and the Netherlands (mean = 3.46, StDev = .33), and Italy (mean = 3.28, StDev = .33) and the Netherlands.

**Table 2:** Results of the ECCQI per country and category, mean and median score 4 point scale and range, *StDev* standard deviation

Category		HUN	PRT	NLD	ROM	LIT	ITA	Total
Accessibility	Mean	3.03	3.39	3.79	2.84	3.03	3.58	3.32
	Median	3.00	3.50	4.00	2.67	3.00	3.67	3.50
	Range	3.00	2.33	3.00	3.00	3.00	2.33	3.00
	StDev	.81	.54	.42	.76	.66	.57	.72
Organization	Mean	2.21	2.13	2.35	2,43	2,32	3,25	2.29
	Median	2.33	2.00	2.20	2.33	2.33	2.17	2.33
	Range	2.33	2.33	2.27	2.00	2.40	2.33	2.60
	StDev	,50	.50	,53	,52	,48	,52	,51
Hospitalization	Mean	3,33	3,36	3,39	3,17	3,23	3,01	3,25
	Median	3.50	3.50	3.50	3.25	3.33	3.00	3.33
	Range	2.00	1.50	1.00	1.67	1.47	2.00	2.33
	StDev	,37	,40	,28	,41	,35	,39	,38
Safety	Mean	3.68	3.93	3.81	3.71	3.61	3.90	3.77
	Median	4.00	4.00	4.00	4.00	3.50	4.00	4.00
	Range	3.00	1.00	2.00	3.00	2.00	1.00	3.00
	StDev	.57	.20	.45	.53	.48	.27	.46
Attitude of HP	Mean	3,39	3,55	3,57	3,69	3,70	3,45	3,55
	Median	3.50	3.67	3.80	3.83	3.92	3.55	3.67
	Range	2.20	2.00	2.00	1.83	2.00	2.20	2.20
	StDev	,57	,52	,48	,41	,47	,49	,51
Communication and information	Mean	3,49	3,68	3,62	3,65	3,56	3,52	3,59
	Median	3.75	4.00	3.75	3.88	3.75	3.67	3.75
	Range	3.00	2.00	2.00	2.00	2.50	2.25	3.00
	StDev	,60	,51	,48	,53	,50	,49	,52
Own input	Mean	3,34	3,11	3,54	3,33	3,45	3,08	3,33
	Median	3.50	3.50	4.00	3.50	4.00	3.00	3.50
	Range	3.00	3.00	2.50	3.00	3.00	3.00	3.00
	StDev	,75	,97	,60	,80	,75	,89	,80
Coordination	Mean	3,46	3,20	3,27	3,43	3,50	3,03	3,31
	Median	3.50	3.25	3.25	3.50	3.50	3.00	3.33
	Range	2.50	2.25	2.75	2.00	2.50	2.50	2.75
	StDev	,49	,59	,62	,52	,48	,54	,57
Supervision and support	Mean	3,26	3,29	3,32	3,20	3,45	3,15	3,28
	Median	3.33	3.40	3.56	3.30	3.60	3.30	3.40
	Range	2.40	2.30	3.00	2.70	2.78	2.33	3.00
	StDev	,58	,62	,75	,61	,57	,63	,63
Rounding off the treatment	Mean	2,99	3,05	3,10	3,23	3,25	3,29	3,11
	Median	3.17	3.25	3.33	3.33	3.33	3.00	3.33
	Range	2.67	1.67	2.33	2.50	2.07	2.50	2.83
	StDev	,53	,58	,63	,57	,40	,61	,54
Mean of all categories	Mean	3,29	3,36	3,46	3,35	3,35	3,28	3,35
	Median	3.37	3.43	3.56	3.40	3.40	3.34	3.41
	Range	1.69	1.42	1.49	1.85	1.85	1.67	1.95
	StDev	,34	,34	.32	,32	,33	,33	,33

Looking more specifically Portugal (mean = 3.11 and StDev = .97) scored fairly low on ‘own inputs’ as does Italy (mean = 3.09 and StDev = .89). ‘Coordination’ is scored quite low by Italian patients (mean = 3.03 and StDev = .54), whereas Hungarian patients give a relatively low score to ‘rounding of the treatment (mean = 2.99 and StDev = .53). However, for none of the categories significant differences were found between highest scoring country and the lowest scoring country. Looking at some specific questions about practical experiences it was found that patients in Hungary, Romania and Lithuania found it difficult to park at the institute (average score of 1). In all countries except Romania the majority of the patients received their diagnosis when expected (in Romania a majority, 47.5 %, received it sooner). For a detailed overview of the outcomes of each separate question see Additional file 1. Looking at the satisfaction questions specifically (Table 3) it can be seen that all patients give a higher grade to the likeliness of recommending the center than to how they experienced the center themselves.

**Table 3:** Overall opinion absolute numbers, mean and median scale 1–10 and range, *StDev* standard deviation

Category		HUN	PRT	NLD	ROM	LIT	ITA
Hospital score	Mean	8,91	8,91	9,11	9,24	8,78	8,57
	Median	9,00	10,00	10,00	10,00	9,00	10,00
	Range	5,00	5,00	3,00	4,00	7,00	6,00
	StDev	1,15	1,27	,87	,98	1,37	1,25
Likeliness to recommend	Mean	9,46	9,42	9,53	9,65	9,02	9,10
	Median	10,00	10,00	10,00	10,00	10,00	10,00
	Range	9,00	7,00	3,00	4,00	7,00	6,00
	StDev	1,19	1,27	,81	,82	1,39	1,12

### Patient characteristics

When looking at the division by age it can be seen that patients who are 65 or older report the highest score at half of all categories. The total scale score increased with age, being 3.27 (StDev = .39) in patients aged 18–34, 3.34 (StDev = .33) in patients 35–64 and 3.39 (StDev = .32) in patients aged >65. The age differences were not significant [F(2692) = 2.68, p = .069]. Stratification by gender shows that females scored lower (mean = 3.34, StDev = .33) compared to males (mean = 3.38, StDev = .34), but this difference is not significant [F(1696) = 1.828, p = 0.177]. Also, quality of care was not reported differently by patients with a higher/longer education [F(5694) = 0.093, p = .911]. When we clustered the patients on tumor type, we observed no significant differences [F(14,683) = 1.297, p = 0.204]. A representative subset of 172 patients (score 1 believing the patient role is important N = 31; score 2 having the confidence and knowledge necessary to take action N = 32; score 3 actually taking action to maintain and improve one’s health N = 76; and score 4 staying the course even under stress N = 33) also completed the PAM, which revealed that reported quality of care significantly differs across PAM level [F(3168) = 2.362, p < 0.034]. Post hoc comparisons showed that this effect is mainly driven by patients in the highest level of activation scoring higher (mean = 3.48, StDev = .26) than respondents with the lowest level (mean = 3.26, StDev = .36) of activation.

### Validity and evaluation of the questions

Fourteen cognitive interviews were conducted. For interviewee characteristics see Additional file 2. Patients felt that in general the questionnaire was appropriate to measure patient satisfaction and experience. However, in 18 questions at least one problem was identified based on the QAS-99 method [19]. Most problems concerned the interpretation of questions. A full overview of the problems can be found in Additional file 3. The most frequently mentioned comment was that the questionnaire does not differentiate between nurses and doctors (N = 7), whereby patients could not give a nuanced answer. CFA revealed that the ECCQI measurement model had a moderate to good fit on our data (RMSEA = 0.039, CFI = 0.943).

### Internal consistency

Seven categories (‘attitude of the healthcare professional’, ‘communication and information’, ‘coordination’, supervision and support’ and ‘rounding off the treatment’) represent a good level of internal consistency (α > 0.7) for all countries and overall (see Table 4). In three categories (’ organization’, ‘hospitalization’ and ‘own inputs) level of internal consistency was acceptable (α between .5 and .7) to good. The alphas in the categories ‘accessibility’ and ‘safety’ were lower and represented an unacceptable internal consistency (α > 0.5) in three countries (accessibility), possibly due to a low number of variables (accessibility = 3, safety = 2) and a smaller sample size after splitting the data to country specific. With the exemption of the Dutch population, removing the question: “Is it difficult to get to the this hospital (either by your own transport, by public transport or by taxi)” could increase α, but the correlational stability of this item increased with sample size.

**Table 4:** Ordinal Cronbach’s alpha(α) score per ECCQI category and country and number of respondents (N) per ECCQI category and country

Category (N items)		HUN	PRT	NLD	ROM	LIT	ITA	Total
Accessibility (3)	α	.37	.23	.73	.54	.49	.71	.70
	Valid N	59	69	120	66	76	95	485
Organization (5)	α	.68	.58	.63	.58	.63	.60	.68
	Valid N	41	27	24	46	48	36	222
Hospitalization (5)	α	.72	.77	.81	.68	.62	.59	.73
	Valid N	88	53	84	87	102	69	483
Safety (2)	α	.64	.43	.66	.78	.54	.90	.65
	Valid N	96	92	95	99	97	107	586
Attitude of HP (6)	α	.91	.91	.91	.91	.93	.86	.91
	Valid N	61	62	31	63	48	82	347
Communication and information (4)	α	.90	.87	.88	.90	.84	.81	.88
	Valid N	102	82	113	96	92	100	585
Own inputs (2)	α	.65	.76	.87	.80	.78	.81	.78
	Valid N	87	60	111	80	76	85	499
Coordination (4)	α	.81	.76	.84	.71	.84	.70	.78
	Valid N	103	86	109	92	93	104	587
Supervision and support (10)	α	.90	.90	.96	.90	.92	.91	.93
	Valid N	40	45	9	37	39	51	221
Rounding off the treatment (4)	α	.82	.95	.77	.86	.96	.93	.78
	Valid N	10	7	11	17	36	14	95

## Discussion

We developed a questionnaire that measures patient experiences and satisfaction with cancer care in hospitals in European countries for patients with all types of cancer. It measures a broad array of topics capturing specific needs and wishes of cancer patients. We found no significant differences between tumor types, supporting the use of a generic questionnaire [4].

With regard to our first question - ‘What are the differences in patient experience and satisfaction between countries and/or patient characteristics?’ we found that patient experience and satisfaction is scored different between countries, with significant differences ranging from an average of 3.27 to 3.46 on a 4-point scale. Patient experience and satisfaction is scored, on average, the lowest in Italy and the highest in the Netherlands. Using one questionnaire for different cultural groups (different nationalities) could lead to measurement bias which could be an explanation for the differences between countries. Looking at possible effects of cultural differences applying Hofstede’s cultural dimension theory [29, 30], possible explanatory factors for the difference in patient satisfaction between countries can be found. High masculine societies (Hungary and Italy) had significantly lower satisfaction scores than low masculine societies (the Netherlands). According to Hofstede, Hofstede & Minkov [31], a high masculine score indicates an assertive judgemental behaviour without having much concern for the feelings of others, which could result in lower satisfaction scores. A low masculine score indicates more tenderness and sympathy for others, resulting in less willingness to provide criticism and therefore higher satisfaction scores. Previous studies on ethnic groups [32, 33] showed however that differences in satisfaction with care should not be ascribed to measurement bias but should be viewed as arising from actual differences in experiences. Evaluation of the measurement equivalence across race and ethnicity on the CAHPS shows that that measurement bias does not substantively influence conclusions based on patients’ responses [33]. A study amongst 15 countries performed by Ipsos [34] showed that Italy scores low on patient experience which corresponds to our findings. Another population survey conducted in 2010 [35] showed a high degree of satisfaction with health-care services and access to health care in both outpatient and inpatient setting in Lithuania.

Regarding the second question: What is the validity and internal consistency (reliability) of the ECCQI?- the cognitive interviews showed problems with different questions. Most problems concerned the interpretation of questions. These questions will be reviewed in order to make them more clear and understandable. The structural validity of the ECCQI measurement model was moderate to good. Given the relative large number of items and scales, versus the number of respondents, the fit could be improved by including more persons to increase the person vs. item ratio. Also, the fit of the model was evaluated for all six countries combined and it is possible that the ECCQI is not measurement invariant across countries or cultures. With more data, it would be possible to investigate whether the measurement model (and thus the latent constructs of the scales) are identical across nations [36]. The validity of the ECCQI could be also be increased with more specificity in the questions, for example by dividing healthcare professionals into doctors and nurses. Regarding internal consistency, alpha was satisfactory to good in eight out of ten categories. Lack of questions in the categories with a low alpha are most likely the reason for the low internal consistency score. It is recommended to investigate whether reliable scales could be created by means of creating other sub-scales, or replace these scales by singe-item questions.

The small differences between countries could be attributed to the difference in completing the questionnaire. In the Netherlands the questionnaires were Internet-based, while in other countries they were paper-based. Studies investigating the equivalence between Internet and paper-based questionnaires are conflicting. Fang [37] indicated that differences were apparent when analyzing data from distinct survey modes (Internet and paper-based). On the other hand, other studies provided results which support the measurement equivalence of survey instruments across Internet and paper-based surveys [38–40].

Age does not significantly influence the results. For the total satisfaction score in all countries, differences between the highest scoring age group and lowest scoring group were not significant. This finding contrasts other studies [41, 42] showing that age needs to be considered when looking at patient experience and satisfaction data. In addition, results show that males were more positive than women which corresponds to results from other studies [41], this difference was however not significant. Further, level of activation seems to have a significant influence, since low activated patients reported lower scores and highly activated patients reported higher scores. It can be seen that all patients give a higher mark to likelihood that they would recommend the hospital to other patients than that they rate the hospitals for themselves. Our results indicate that when measuring patient experience and satisfaction results need to be adjusted for nationality and level of activation but not for age or other demographic characteristics. Based on this research, the current questionnaire should be further tested for its ability to discriminate between hospitals and countries.

A possible limitation of this study design is the sampling method. With convenience sampling the chance of selection bias is high which could have influenced the outcomes. For example, in education level a majority of the Portuguese patients had a low education level, a majority of the Italian patient had a moderate education while in the other countries the majority had a high education level. Regarding physical health, patients in Portugal were more negative giving a moderate score, while in the other countries most patients rated their physical health as good or excellent. Analysis of the total study population however showed no influence of demographic characteristics.

The real value of these studies lies in their use to stimulate quality improvements. Even though the centers studied are not necessarily representative of all cancer centers in the study countries, the results indicate areas of improvement and might provide evidence about how organizations and providers could meet patients’ needs more effectively.

## Conclusions

To our knowledge, the questionnaire used in this study is the first that measures the experiences and satisfaction of cancer patients with care provided by cancer centers in Europe. Our results show that patient satisfaction is scored significantly different between countries. We showed that differences exist in experiences and satisfaction between people with different characteristics such as activation levels. After testing for discriminatory power our questionnaire can be used Europe-wide to measure quality of cancer care from the patient perspective and to identify differences in the experiences of patients in different hospitals. This ECCQI is a first step towards the international comparison of patient experience and satisfaction, which could enable healthcare providers and policy makers to improve the quality of cancer care.

## Additional files

Additional file 1: Overview of results per question and country. This files gives the answers to each question per country, both in absolute numbers (how many patients gave that specific answer) and percentage. (DOCX 103 kb) Additional file 2: Interviewee characteristics. This file describes the characteristics of the participant to the cognitive interviews. (DOCX 14 kb) Additional file 3: Problems per ECCQI-question. This file gives an overview of the problems identified per question during the cognitive interviews and through feedback on the questionnaire. (DOCX 20 kb)

## Abbreviations

ANOVA: Analyses of variance
CAHPS: Consumer assessment of healthcare providers and systems
CFA: Confirmatory factor analysis
CFI: Comparative fit index
CI: Confidence interval
CQI: Consumer quality index
ECCQI: European cancer consumer quality index
HUN: Hungary
IOM: Institute of medicine
ITA: Italy
LIT: Lithuania
NLD: the Netherlands
PAM: Patient activation measure
PRT: Portugal
QAS: Question appraisal system
QUOTE: Quality of care through the patient’s eyes
RMSEA: Root mean square error of approximation
ROM: Romania
StDev: Standard deviation
WLSMV: Weighted least squares mean variance
