# Supplementary material for: Piloting a generic cancer consumer quality index in six European countries
Source: BMC Cancer. 2016 Sep 2;16(1):711. doi: 10.1186/s12885-016-2752-9 (PMC5010728; doi:10.1186/s12885-016-2752-9)
Supplement: Additional file 1: — Overview of results per question and country. This files gives the answers to each question per country, both in absolute numbers (how many patients gave that specific answer) and percentage. (DOCX 103 kb) [file 12885_2016_2752_MOESM1_ESM.docx]

Additional file 1. Overview of results per question and country. Grouped by category

Category 1 Accessibility

|  | | | Nationality | | | | | |
| --- | --- | --- | --- | --- | --- | --- | --- | --- |
|  |  |  | HUN | PRT | NLD | ROM | LIT | ITA |
| Was it difficult to get to the this hospital (either by your own transport, by public transport or by taxi)? | Very difficult | N | 11 | 5 | 2 | 9 | 3 | 2 |
|  |  | % | 8,9% | 5,3% | 1,4% | 9,0% | 2,7% | 1,8% |
|  | Not very difficult | N | 34 | 26 | 17 | 41 | 36 | 23 |
|  |  | % | 27,6% | 27,4% | 11,5% | 41,0% | 32,1% | 20,2% |
|  | Not at all difficult | N | 78 | 64 | 129 | 50 | 73 | 89 |
|  |  | % | 63,4% | 67,4% | 87,2% | 50,0% | 65,2% | 78,1% |
| Was it difficult to park at this hospital? | Very difficult | N | 36 | 20 | 5 | 47 | 43 | 16 |
|  |  | % | 54,5% | 24,7% | 3,5% | 62,7% | 49,4% | 15,1% |
|  | Not very difficult | N | 19 | 31 | 24 | 22 | 36 | 22 |
|  |  | % | 28,8% | 38,3% | 17,0% | 29,3% | 41,4% | 20,8% |
|  | Not at all difficult | N | 11 | 30 | 112 | 6 | 8 | 68 |
|  |  | % | 16,7% | 37,0% | 79,4% | 8,0% | 9,2% | 64,2% |
| Was it difficult to reach this hospital by phone? | Very difficult | N | 25 | 1 | 2 | 13 | 12 | 3 |
|  |  | % | 23,1% | 1,3% | 1,6% | 15,3% | 12,5% | 2,9% |
|  | Not very difficult | N | 36 | 24 | 18 | 28 | 40 | 28 |
|  |  | % | 33,3% | 30,4% | 14,1% | 32,9% | 41,7% | 27,2% |
|  | Not at all difficult | N | 47 | 54 | 108 | 44 | 44 | 72 |
|  |  | % | 43,5% | 68,4% | 84,4% | 51,8% | 45,8% | 69,9% |

Category 2 Organization

|  | | | Nationality | | | | | |
| --- | --- | --- | --- | --- | --- | --- | --- | --- |
|  |  |  | HUN | PRT | NLD | ROM | LIT | ITA |
| How long did it last between your referral to the hospital and your first visit there? | More than 15 weekdays | N | 20 | 9 | 2 | 10 | 17 | 4 |
|  |  | % | 35,7% | 26,5% | 4,1% | 17,2% | 24,3% | 9,8% |
|  | 11-15 weekdays | N | 14 | 6 | 12 | 7 | 11 | 2 |
|  |  | % | 25,0% | 17,6% | 24,5% | 12,1% | 15,7% | 4,9% |
|  | 6-10 weekdays | N | 8 | 9 | 18 | 12 | 25 | 14 |
|  |  | % | 14,3% | 26,5% | 36,7% | 20,7% | 35,7% | 34,1% |
|  | Less than 6 weekdays | N | 14 | 10 | 17 | 29 | 17 | 21 |
|  |  | % | 25,0% | 29,4% | 34,7% | 50,0% | 24,3% | 51,2% |
| How long did it last between your first visit/examination and your diagnosis? | More than 15 weekdays | N | 22 | 8 | 5 | 18 | 23 | 9 |
|  |  | % | 37,9% | 22,9% | 9,4% | 31,6% | 31,5% | 21,4% |
|  | 11-15 weekdays | N | 17 | 9 | 8 | 13 | 25 | 6 |
|  |  | % | 29,3% | 25,7% | 15,1% | 22,8% | 34,2% | 14,3% |
|  | 6-10 weekdays | N | 11 | 11 | 11 | 5 | 16 | 9 |
|  |  | % | 19,0% | 31,4% | 20,8% | 8,8% | 21,9% | 21,4% |
|  | Less than 6 weekdays | N | 8 | 7 | 29 | 21 | 9 | 18 |
|  |  | % | 13,8% | 20,0% | 54,7% | 36,8% | 12,3% | 42,9% |
| Did you hear the diagnosis sooner or later than you had expected? | Much later | N | 3 | 1 | 2 | 0 | 3 | 2 |
|  |  | % | 4,7% | 2,9% | 3,5% | 0,0% | 3,8% | 4,4% |
|  | Later | N | 8 | 3 | 2 | 12 | 9 | 8 |
|  |  | % | 12,5% | 8,6% | 3,5% | 18,8% | 11,3% | 17,8% |
|  | When I’d expected it | N | 36 | 24 | 34 | 23 | 57 | 19 |
|  |  | % | 56,3% | 68,6% | 59,6% | 35,9% | 71,3% | 42,2% |
|  | Sooner or much sooner | N | 17 | 7 | 19 | 29 | 11 | 16 |
|  |  | % | 26,6% | 20,0% | 33,3% | 45,3% | 13,8% | 35,6% |
| Once the diagnosis was known, was it possible to start treatment as quickly as you wanted? | No | N | 19 | 14 | 19 | 8 | 16 | 6 |
|  |  | % | 16,7% | 15,7% | 17,1% | 7,9% | 16,0% | 5,7% |
|  | Yes | N | 95 | 75 | 92 | 93 | 84 | 99 |
|  |  | % | 83,3% | 84,3% | 82,9% | 92,1% | 84,0% | 94,3% |
| If you desired this, was it possible at this hospital to plan several appointments for examination and/or treatment on the same day? | Never | N | 5 | 7 | 0 | 5 | 5 | 9 |
|  |  | % | 5,9% | 8,9% | 0,0% | 6,2% | 5,4% | 8,7% |
|  | Sometimes | N | 14 | 31 | 20 | 25 | 20 | 24 |
|  |  | % | 16,5% | 39,2% | 14,5% | 30,9% | 21,5% | 23,3% |
|  | Usually | N | 31 | 22 | 72 | 19 | 42 | 54 |
|  |  | % | 36,5% | 27,8% | 52,2% | 23,5% | 45,2% | 52,4% |
|  | Always | N | 35 | 19 | 46 | 32 | 26 | 16 |
|  |  | % | 41,2% | 24,1% | 33,3% | 39,5% | 28,0% | 15,5% |

Category 3 Hospitalization

|  | | | Nationality | | | | | |
| --- | --- | --- | --- | --- | --- | --- | --- | --- |
|  |  |  | HUN | PRT | NLD | ROM | LIT | ITA |
| Were the toilet, shower and bathroom in or near the room? | Never | N | 1 | 2 | 0 | 1 | 14 | 0 |
|  |  | % | 1,1% | 3,8% | 0,0% | 1,1% | 13,0% | 0,0% |
|  | Sometimes | N | 1 | 4 | 1 | 6 | 29 | 1 |
|  |  | % | 1,1% | 7,5% | 1,0% | 6,8% | 26,9% | 1,4% |
|  | Usually | N | 5 | 7 | 5 | 21 | 37 | 10 |
|  |  | % | 5,4% | 13,2% | 5,0% | 23,9% | 34,3% | 14,1% |
|  | Always | N | 85 | 40 | 95 | 60 | 28 | 60 |
|  |  | % | 92,4% | 75,5% | 94,1% | 68,2% | 25,9% | 84,5% |
| Was your privacy sufficiently respected at this hospital (when changing clothes, washing/showering, during visiting hours, no information given in the presence of other patients)? | Never | N | 8 | 1 | 2 | 3 | 1 | 5 |
|  |  | % | 8,7% | 1,9% | 2,1% | 3,4% | 0,9% | 7,0% |
|  | Sometimes | N | 3 | 4 | 1 | 6 | 6 | 2 |
|  |  | % | 3,3% | 7,5% | 1,0% | 6,8% | 5,5% | 2,8% |
|  | Usually | N | 12 | 6 | 22 | 25 | 13 | 23 |
|  |  | % | 13,0% | 11,3% | 22,7% | 28,4% | 11,9% | 32,4% |
|  | Always | N | 69 | 42 | 72 | 54 | 89 | 41 |
|  |  | % | 75,0% | 79,2% | 74,2% | 61,4% | 81,7% | 57,7% |
| Were you able to receive visitors at the times you wanted? | Never | N | 1 | 1 | 1 | 0 | 0 | 5 |
|  |  | % | 1,1% | 1,9% | 1,0% | 0,0% | 0,0% | 7,1% |
|  | Sometimes | N | 2 | 1 | 1 | 8 | 0 | 13 |
|  |  | % | 2,2% | 1,9% | 1,0% | 9,2% | 0,0% | 18,6% |
|  | Usually | N | 12 | 9 | 18 | 29 | 18 | 32 |
|  |  | % | 13,0% | 17,0% | 18,4% | 33,3% | 16,4% | 45,7% |
|  | Always | N | 77 | 42 | 78 | 50 | 92 | 20 |
|  |  | % | 83,7% | 79,2% | 79,6% | 57,5% | 83,6% | 28,6% |
| Were you able to be undisturbed whenever you wished? | Never | N | 5 | 2 | 0 | 9 | 4 | 2 |
|  |  | % | 5,6% | 3,8% | 0,0% | 10,2% | 3,7% | 2,8% |
|  | Sometimes | N | 13 | 5 | 8 | 17 | 9 | 7 |
|  |  | % | 14,4% | 9,4% | 8,6% | 19,3% | 8,3% | 9,9% |
|  | Usually | N | 36 | 10 | 30 | 28 | 35 | 40 |
|  |  | % | 40,0% | 18,9% | 32,3% | 31,8% | 32,1% | 56,3% |
|  | Always | N | 36 | 36 | 55 | 34 | 61 | 22 |
|  |  | % | 40,0% | 67,9% | 59,1% | 38,6% | 56,0% | 31,0% |
| Was it possible to eat at the times you wished? | Never | N | 1 | 0 | 2 | 0 | 1 | 8 |
|  |  | % | 1,1% | 0,0% | 2,1% | 0,0% | 0,9% | 11,6% |
|  | Sometimes | N | 6 | 7 | 7 | 8 | 5 | 15 |
|  |  | % | 6,5% | 13,2% | 7,4% | 9,1% | 4,7% | 21,7% |
|  | Usually | N | 22 | 6 | 27 | 29 | 24 | 36 |
|  |  | % | 23,9% | 11,3% | 28,7% | 33,0% | 22,4% | 52,2% |
|  | Always | N | 63 | 40 | 58 | 51 | 77 | 10 |
|  |  | % | 68,5% | 75,5% | 61,7% | 58,0% | 72,0% | 14,5% |

Category 4 Safety

|  | | | Nationality | | | | | |
| --- | --- | --- | --- | --- | --- | --- | --- | --- |
|  |  |  | HUN | PRT | NLD | ROM | LIT | ITA |
| When you were being given medicine, did anyone check that it was really intended for you – by asking your name, for example, or checking your hospital wristband? | Never | N | 2 | 0 | 4 | 1 | 5 | 0 |
|  |  | % | 2,0% | 0,0% | 4,2% | 1,0% | 5,2% | 0,0% |
|  | Sometimes | N | 3 | 2 | 4 | 2 | 9 | 0 |
|  |  | % | 3,1% | 2,2% | 4,2% | 2,0% | 9,3% | 0,0% |
|  | Usually | N | 26 | 5 | 12 | 19 | 42 | 14 |
|  |  | % | 26,5% | 5,4% | 12,6% | 19,2% | 43,3% | 12,8% |
|  | Always | N | 67 | 85 | 75 | 77 | 41 | 95 |
|  |  | % | 68,4% | 92,4% | 78,9% | 77,8% | 42,3% | 87,2% |
| Before treatment, examination or an operation began, did anyone check that you were the right person – by asking your name and date of birth, for example? | Never | N | 2 | 0 | 0 | 2 | 0 | 0 |
|  |  | % | 1,7% | 0,0% | 0,0% | 2,0% | 0,0% | 0,0% |
|  | Sometimes | N | 3 | 0 | 3 | 4 | 2 | 0 |
|  |  | % | 2,5% | 0,0% | 2,1% | 3,9% | 1,8% | 0,0% |
|  | Usually | N | 17 | 4 | 6 | 16 | 7 | 7 |
|  |  | % | 14,4% | 4,2% | 4,1% | 15,7% | 6,4% | 6,4% |
|  | Always | N | 96 | 91 | 136 | 80 | 100 | 103 |
|  |  | % | 81,4% | 95,8% | 93,8% | 78,4% | 91,7% | 93,6% |

Category 5 Attitude of HP

|  | | | Nationality | | | | | |
| --- | --- | --- | --- | --- | --- | --- | --- | --- |
|  |  |  | HUN | PRT | NLD | ROM | LIT | ITA |
| Did the healthcare professionals listen to you attentively? | No, none of them did | N | 1 | 0 | 0 | 0 | 0 | 0 |
|  |  | % | 0,8% | 0,0% | 0,0% | 0,0% | 0,0% | 0,0% |
|  | Some of them did | N | 4 | 5 | 3 | 4 | 5 | 6 |
|  |  | % | 3,2% | 5,2% | 2,0% | 4,1% | 4,5% | 5,3% |
|  | Most of them did | N | 49 | 25 | 41 | 18 | 19 | 38 |
|  |  | % | 39,5% | 26,0% | 27,9% | 18,4% | 17,0% | 33,6% |
|  | Yes, all of them did | N | 70 | 66 | 103 | 76 | 88 | 69 |
|  |  | % | 56,5% | 68,8% | 70,1% | 77,6% | 78,6% | 61,1% |
| Did the healthcare professionals have enough time for you? | No, none of them did | N | 0 | 0 | 0 | 0 | 0 | 0 |
|  |  | % | 0,0% | 0,0% | 0,0% | 0,0% | 0,0% | 0,0% |
|  | Some of them did | N | 10 | 9 | 3 | 5 | 6 | 15 |
|  |  | % | 8,1% | 9,4% | 2,0% | 5,1% | 5,4% | 13,2% |
|  | Most of them did | N | 49 | 33 | 46 | 27 | 26 | 45 |
|  |  | % | 39,5% | 34,4% | 31,1% | 27,6% | 23,2% | 39,5% |
|  | Yes, all of them did | N | 65 | 54 | 99 | 66 | 80 | 54 |
|  |  | % | 52,4% | 56,3% | 66,9% | 67,3% | 71,4% | 47,4% |
| Did the healthcare professionals take you seriously? | No, none of them did | N | 0 | 0 | 1 | 0 | 0 | 0 |
|  |  | % | 0,0% | 0,0% | 0,7% | 0,0% | 0,0% | 0,0% |
|  | Some of them did | N | 5 | 4 | 2 | 2 | 5 | 4 |
|  |  | % | 4,0% | 4,2% | 1,4% | 2,0% | 4,5% | 3,5% |
|  | Most of them did | N | 34 | 16 | 29 | 15 | 13 | 24 |
|  |  | % | 27,4% | 16,7% | 19,6% | 15,3% | 11,7% | 21,1% |
|  | Yes, all of them did | N | 85 | 76 | 116 | 81 | 93 | 86 |
|  |  | % | 68,5% | 79,2% | 78,4% | 82,7% | 83,8% | 75,4% |
| Were there opportunities to talk with your healthcare professionals about how you felt? | Never | N | 2 | 1 | 2 | 0 | 0 | 3 |
|  |  | % | 1,6% | 1,0% | 1,4% | 0,0% | 0,0% | 2,7% |
|  | Sometimes | N | 17 | 15 | 15 | 5 | 6 | 9 |
|  |  | % | 13,8% | 15,6% | 10,3% | 5,1% | 5,4% | 8,0% |
|  | Usually | N | 51 | 14 | 45 | 22 | 19 | 45 |
|  |  | % | 41,5% | 14,6% | 31,0% | 22,4% | 17,0% | 39,8% |
|  | Always | N | 53 | 66 | 83 | 71 | 87 | 56 |
|  |  | % | 43,1% | 68,8% | 57,2% | 72,4% | 77,7% | 49,6% |
| Did your healthcare professionals pay attention to your loved one(s)? | No, none of them did | N | 14 | 4 | 2 | 1 | 2 | 4 |
|  |  | % | 12,4% | 4,2% | 1,5% | 1,1% | 1,8% | 3,6% |
|  | Some of them did | N | 17 | 19 | 18 | 5 | 5 | 23 |
|  |  | % | 15,0% | 19,8% | 13,3% | 5,3% | 4,6% | 20,5% |
|  | Most of them did | N | 45 | 24 | 50 | 31 | 22 | 40 |
|  |  | % | 39,8% | 25,0% | 37,0% | 32,6% | 20,2% | 35,7% |
|  | Yes, all of them did | N | 37 | 49 | 65 | 58 | 80 | 45 |
|  |  | % | 32,7% | 51,0% | 48,1% | 61,1% | 73,4% | 40,2% |
| Did your healthcare professionals show due respect to faith or philosophy of life? | No, none of them did | N | 2 | 2 | 1 | 0 | 1 | 1 |
|  |  | % | 3,1% | 3,2% | 3,0% | 0,0% | 2,0% | 1,2% |
|  | Some of them did | N | 0 | 1 | 0 | 2 | 5 | 6 |
|  |  | % | 0,0% | 1,6% | 0,0% | 3,2% | 10,0% | 7,2% |
|  | Most of them did | N | 17 | 9 | 11 | 3 | 9 | 11 |
|  |  | % | 26,6% | 14,5% | 33,3% | 4,8% | 18,0% | 13,3% |
|  | Yes, all of them did | N | 45 | 50 | 21 | 58 | 35 | 65 |
|  |  | % | 70,3% | 80,6% | 63,6% | 92,1% | 70,0% | 78,3% |

Category 6 Communication and information

|  | | | Nationality | | | | | |
| --- | --- | --- | --- | --- | --- | --- | --- | --- |
|  |  |  | HUN | PRT | NLD | ROM | LIT | ITA |
| Did healthcare professionals explain things to you in ways that were clear and understandable? | Never | N | 3 | 0 | 1 | 0 | 1 | 0 |
|  |  | % | 2,4% | 0,0% | 0,7% | 0,0% | 0,9% | 0,0% |
|  | Sometimes | N | 7 | 3 | 2 | 8 | 4 | 3 |
|  |  | % | 5,7% | 3,1% | 1,4% | 7,8% | 3,6% | 2,7% |
|  | Usually | N | 40 | 15 | 39 | 20 | 24 | 46 |
|  |  | % | 32,5% | 15,6% | 26,4% | 19,6% | 21,4% | 40,7% |
|  | Always | N | 73 | 78 | 106 | 74 | 83 | 64 |
|  |  | % | 59,3% | 81,3% | 71,6% | 72,5% | 74,1% | 56,6% |
| Did the healthcare professionals give you information about any side-effects of the treatment? | Never | N | 3 | 1 | 1 | 0 | 2 | 1 |
|  |  | % | 2,7% | 1,1% | 0,8% | 0,0% | 2,0% | 0,9% |
|  | Sometimes | N | 9 | 5 | 9 | 5 | 8 | 4 |
|  |  | % | 8,0% | 5,3% | 6,9% | 5,0% | 7,8% | 3,6% |
|  | Usually | N | 28 | 9 | 33 | 18 | 22 | 25 |
|  |  | % | 25,0% | 9,6% | 25,2% | 18,0% | 21,6% | 22,7% |
|  | Always | N | 72 | 79 | 88 | 77 | 70 | 80 |
|  |  | % | 64,3% | 84,0% | 67,2% | 77,0% | 68,6% | 72,7% |
| During your treatment, were you informed about its effect ((for example whether you were responding to it)? | Never | N | 2 | 2 | 1 | 1 | 1 | 4 |
|  |  | % | 1,8% | 2,3% | 0,8% | 1,0% | 1,0% | 3,8% |
|  | Sometimes | N | 7 | 9 | 12 | 8 | 7 | 7 |
|  |  | % | 6,4% | 10,2% | 9,8% | 7,9% | 6,9% | 6,7% |
|  | Usually | N | 29 | 12 | 26 | 19 | 26 | 28 |
|  |  | % | 26,6% | 13,6% | 21,3% | 18,8% | 25,5% | 26,9% |
|  | Always | N | 71 | 65 | 83 | 73 | 68 | 65 |
|  |  | % | 65,1% | 73,9% | 68,0% | 72,3% | 66,7% | 62,5% |
| Was the written information about the examinations or treatment clear? | Never | N | 2 | 2 | 1 | 0 | 0 | 3 |
|  |  | % | 1,8% | 2,3% | 0,7% | 0,0% | 0,0% | 2,8% |
|  | Sometimes | N | 10 | 11 | 2 | 7 | 8 | 6 |
|  |  | % | 8,8% | 12,8% | 1,4% | 7,1% | 7,8% | 5,6% |
|  | Usually | N | 41 | 13 | 42 | 23 | 42 | 42 |
|  |  | % | 36,3% | 15,1% | 29,8% | 23,2% | 40,8% | 38,9% |
|  | Always | N | 60 | 60 | 96 | 69 | 53 | 57 |
|  |  | % | 53,1% | 69,8% | 68,1% | 69,7% | 51,5% | 52,8% |

Category 7 Own inputs

|  | | | Nationality | | | | | |
| --- | --- | --- | --- | --- | --- | --- | --- | --- |
|  |  |  | HUN | PRT | NLD | ROM | LIT | ITA |
| If you wanted, could you take part in decisions about the care and treatment you received? | Never | N | 4 | 5 | 1 | 3 | 2 | 8 |
|  |  | % | 3,6% | 7,6% | 0,8% | 3,4% | 2,2% | 8,9% |
|  | Sometimes | N | 8 | 15 | 8 | 9 | 5 | 11 |
|  |  | % | 7,3% | 22,7% | 6,2% | 10,3% | 5,4% | 12,2% |
|  | Usually | N | 34 | 17 | 42 | 21 | 24 | 35 |
|  |  | % | 30,9% | 25,8% | 32,3% | 24,1% | 25,8% | 38,9% |
|  | Always | N | 64 | 29 | 79 | 54 | 62 | 36 |
|  |  | % | 58,2% | 43,9% | 60,8% | 62,1% | 66,7% | 40,0% |
| Was it possible for loved ones to be involved in discussions on your care and treatment? | Never | N | 9 | 8 | 1 | 4 | 7 | 9 |
|  |  | % | 9,8% | 10,8% | 0,9% | 4,3% | 8,6% | 9,1% |
|  | Sometimes | N | 13 | 9 | 8 | 20 | 6 | 17 |
|  |  | % | 14,1% | 12,2% | 7,1% | 21,5% | 7,4% | 17,2% |
|  | Usually | N | 28 | 13 | 30 | 21 | 23 | 27 |
|  |  | % | 30,4% | 17,6% | 26,5% | 22,6% | 28,4% | 27,3% |
|  | Always | N | 42 | 44 | 74 | 48 | 45 | 46 |
|  |  | % | 45,7% | 59,5% | 65,5% | 51,6% | 55,6% | 46,5% |

Category 8 Coordination

|  | | | Nationality | | | | | |
| --- | --- | --- | --- | --- | --- | --- | --- | --- |
|  |  |  | HUN | PRT | NLD | ROM | LIT | ITA |
| Were the treatment and examinations you had from different healthcare professionals well coordinated? | Never | N | 0 | 0 | 2 | 2 | 1 | 0 |
|  |  | % | 0,0% | 0,0% | 1,4% | 2,1% | 0,9% | 0,0% |
|  | Sometimes | N | 6 | 5 | 8 | 10 | 3 | 7 |
|  |  | % | 5,0% | 5,4% | 5,8% | 10,6% | 2,8% | 6,2% |
|  | Usually | N | 56 | 19 | 46 | 27 | 25 | 56 |
|  |  | % | 46,3% | 20,7% | 33,1% | 28,7% | 22,9% | 49,6% |
|  | Always | N | 59 | 68 | 83 | 55 | 80 | 50 |
|  |  | % | 48,8% | 73,9% | 59,7% | 58,5% | 73,4% | 44,2% |
| Were your healthcare professionals aware of the appointments you had with other healthcare professionals? | Never | N | 5 | 0 | 2 | 3 | 0 | 3 |
|  |  | % | 4,7% | 0,0% | 1,6% | 3,1% | 0,0% | 2,8% |
|  | Sometimes | N | 7 | 9 | 8 | 7 | 6 | 9 |
|  |  | % | 6,5% | 9,7% | 6,2% | 7,2% | 5,9% | 8,3% |
|  | Usually | N | 49 | 22 | 47 | 25 | 31 | 52 |
|  |  | % | 45,8% | 23,7% | 36,4% | 25,8% | 30,7% | 48,1% |
|  | Always | N | 46 | 62 | 72 | 62 | 64 | 44 |
|  |  | % | 43,0% | 66,7% | 55,8% | 63,9% | 63,4% | 40,7% |
| Did you always deal with the same person in this hospital – such as a doctor or nurse – when anything needed to be arranged (planning and coordinating appointments, for example)? | Never | N | 1 | 6 | 12 | 3 | 1 | 10 |
|  |  | % | 0,8% | 6,6% | 9,6% | 3,0% | 1,0% | 8,8% |
|  | Sometimes | N | 3 | 35 | 18 | 10 | 6 | 37 |
|  |  | % | 2,5% | 38,5% | 14,4% | 10,0% | 5,9% | 32,7% |
|  | Usually | N | 45 | 32 | 40 | 31 | 50 | 52 |
|  |  | % | 37,2% | 35,2% | 32,0% | 31,0% | 49,0% | 46,0% |
|  | Always | N | 72 | 18 | 55 | 56 | 45 | 14 |
|  |  | % | 59,5% | 19,8% | 44,0% | 56,0% | 44,1% | 12,4% |
| Were you seen by the same care providers during your investigations and treatments? | Never | N | 1 | 6 | 3 | 0 | 1 | 5 |
|  |  | % | 0,8% | 6,5% | 2,1% | 0,0% | 0,9% | 4,6% |
|  | Sometimes | N | 6 | 30 | 28 | 12 | 2 | 31 |
|  |  | % | 5,0% | 32,6% | 19,6% | 12,0% | 1,8% | 28,4% |
|  | Usually | N | 45 | 30 | 75 | 40 | 59 | 48 |
|  |  | % | 37,8% | 32,6% | 52,4% | 40,0% | 52,7% | 44,0% |
|  | Always | N | 67 | 26 | 37 | 48 | 50 | 25 |
|  |  | % | 56,3% | 28,3% | 25,9% | 48,0% | 44,6% | 22,9% |

Category 9 Supervision and support

|  | | | Nationality | | | | | |
| --- | --- | --- | --- | --- | --- | --- | --- | --- |
|  |  |  | HUN | PRT | NLD | ROM | LIT | ITA |
| During the diagnostic phase, was attention paid to your pain? | Never | N | 0 | 1 | 3 | 0 | 1 | 0 |
|  |  | % | 0,0% | 1,1% | 3,7% | 0,0% | 0,9% | 0,0% |
|  | Sometimes | N | 3 | 3 | 7 | 5 | 2 | 10 |
|  |  | % | 2,9% | 3,4% | 8,6% | 5,5% | 1,8% | 10,1% |
|  | Usually | N | 27 | 16 | 9 | 14 | 21 | 33 |
|  |  | % | 26,5% | 18,4% | 11,1% | 15,4% | 19,3% | 33,3% |
|  | Always | N | 72 | 67 | 62 | 72 | 85 | 56 |
|  |  | % | 70,6% | 77,0% | 76,5% | 79,1% | 78,0% | 56,6% |
| During the treatment phase, was attention paid to your pain? | Never | N | 2 | 0 | 1 | 0 | 0 | 1 |
|  |  | % | 2,0% | 0,0% | 1,0% | 0,0% | 0,0% | 1,0% |
|  | Sometimes | N | 2 | 4 | 3 | 4 | 2 | 5 |
|  |  | % | 2,0% | 4,4% | 2,9% | 4,1% | 1,8% | 5,1% |
|  | Usually | N | 26 | 9 | 22 | 9 | 12 | 30 |
|  |  | % | 26,0% | 10,0% | 21,2% | 9,3% | 10,8% | 30,3% |
|  | Always | N | 70 | 77 | 78 | 84 | 97 | 63 |
|  |  | % | 70,0% | 85,6% | 75,0% | 86,6% | 87,4% | 63,6% |
| During aftercare, was attention paid to your pain? | Never | N | 0 | 0 | 1 | 0 | 2 | 2 |
|  |  | % | 0,0% | 0,0% | 1,0% | 0,0% | 2,0% | 2,2% |
|  | Sometimes | N | 3 | 4 | 7 | 5 | 4 | 7 |
|  |  | % | 3,5% | 5,1% | 7,0% | 6,2% | 4,0% | 7,7% |
|  | Usually | N | 29 | 6 | 22 | 24 | 22 | 29 |
|  |  | % | 34,1% | 7,7% | 22,0% | 29,6% | 22,2% | 31,9% |
|  | Always | N | 53 | 68 | 70 | 52 | 71 | 53 |
|  |  | % | 62,4% | 87,2% | 70,0% | 64,2% | 71,7% | 58,2% |
| During the diagnostic phase, was attention paid to your complaints about fatigue? | Never | N | 4 | 3 | 3 | 2 | 2 | 0 |
|  |  | % | 4,7% | 3,5% | 5,5% | 2,3% | 1,9% | 0,0% |
|  | Sometimes | N | 4 | 9 | 5 | 8 | 5 | 11 |
|  |  | % | 4,7% | 10,6% | 9,1% | 9,3% | 4,6% | 11,7% |
|  | Usually | N | 29 | 13 | 16 | 18 | 20 | 32 |
|  |  | % | 34,1% | 15,3% | 29,1% | 20,9% | 18,5% | 34,0% |
|  | Always | N | 48 | 60 | 31 | 58 | 81 | 51 |
|  |  | % | 56,5% | 70,6% | 56,4% | 67,4% | 75,0% | 54,3% |
| During the treatment phase, was attention paid to your complaints about fatigue? | Never | N | 2 | 1 | 4 | 2 | 1 | 0 |
|  |  | % | 2,4% | 1,1% | 5,2% | 2,2% | 1,1% | 0,0% |
|  | Sometimes | N | 3 | 7 | 6 | 7 | 6 | 9 |
|  |  | % | 3,6% | 8,0% | 7,8% | 7,9% | 6,4% | 8,9% |
|  | Usually | N | 27 | 12 | 17 | 23 | 11 | 30 |
|  |  | % | 32,1% | 13,6% | 22,1% | 25,8% | 11,7% | 29,7% |
|  | Always | N | 52 | 68 | 50 | 57 | 76 | 62 |
|  |  | % | 61,9% | 77,3% | 64,9% | 64,0% | 80,9% | 61,4% |
| During the aftercare, was attention paid to your complaints about fatigue? | Never | N | 2 | 3 | 3 | 3 | 1 | 2 |
|  |  | % | 2,7% | 4,1% | 4,0% | 4,5% | 1,8% | 2,4% |
|  | Sometimes | N | 4 | 7 | 11 | 8 | 6 | 0 |
|  |  | % | 5,4% | 9,5% | 14,7% | 11,9% | 10,5% | 0,0% |
|  | Usually | N | 22 | 14 | 18 | 22 | 14 | 33 |
|  |  | % | 29,7% | 18,9% | 24,0% | 32,8% | 24,6% | 39,8% |
|  | Always | N | 46 | 50 | 43 | 34 | 36 | 48 |
|  |  | % | 62,2% | 67,6% | 57,3% | 50,7% | 63,2% | 57,8% |
| Did this hospital provide you with information about help with coping with emotions and other forms of counselling on this? | Never | N | 2 | 3 | 3 | 3 | 1 | 2 |
|  |  | % | 2,7% | 4,1% | 4,0% | 4,5% | 1,8% | 2,4% |
|  | Sometimes | N | 4 | 7 | 11 | 8 | 6 | 0 |
|  |  | % | 5,4% | 9,5% | 14,7% | 11,9% | 10,5% | 0,0% |
|  | Usually | N | 22 | 14 | 18 | 22 | 14 | 33 |
|  |  | % | 29,7% | 18,9% | 24,0% | 32,8% | 24,6% | 39,8% |
|  | Always | N | 46 | 50 | 43 | 34 | 36 | 48 |
|  |  | % | 62,2% | 67,6% | 57,3% | 50,7% | 63,2% | 57,8% |
| Did this hospital provide you with information about help with dealing with practical problems caused by cancer and other forms of counselling on this? | Never | N | 19 | 11 | 12 | 16 | 3 | 19 |
|  |  | % | 19,2% | 13,1% | 11,5% | 19,3% | 3,3% | 20,2% |
|  | Sometimes | N | 17 | 15 | 23 | 15 | 10 | 15 |
|  |  | % | 17,2% | 17,9% | 22,1% | 18,1% | 11,0% | 16,0% |
|  | Usually | N | 37 | 12 | 16 | 21 | 30 | 25 |
|  |  | % | 37,4% | 14,3% | 15,4% | 25,3% | 33,0% | 26,6% |
|  | Always | N | 26 | 46 | 53 | 31 | 48 | 35 |
|  |  | % | 26,3% | 54,8% | 51,0% | 37,3% | 52,7% | 37,2% |
| Did healthcare professionals inform you about patient organisations? | Never | N | 32 | 35 | 25 | 33 | 22 | 32 |
|  |  | % | 31,7% | 50,7% | 25,8% | 48,5% | 28,9% | 36,8% |
|  | Sometimes | N | 24 | 9 | 18 | 15 | 10 | 23 |
|  |  | % | 23,8% | 13,0% | 18,6% | 22,1% | 13,2% | 26,4% |
|  | Usually | N | 25 | 7 | 21 | 8 | 21 | 11 |
|  |  | % | 24,8% | 10,1% | 21,6% | 11,8% | 27,6% | 12,6% |
|  | Always | N | 20 | 18 | 33 | 12 | 23 | 21 |
|  |  | % | 19,8% | 26,1% | 34,0% | 17,6% | 30,3% | 24,1% |
| Was it possible to talk to a spiritual or moral counsellor, such as a hospital chaplain or humanistic counsellor? | Never | N | 6 | 37 | 4 | 21 | 21 | 23 |
|  |  | % | 7,8% | 56,1% | 12,5% | 22,6% | 21,2% | 25,6% |
|  | Sometimes | N | 9 | 11 | 5 | 24 | 17 | 31 |
|  |  | % | 11,7% | 16,7% | 15,6% | 25,8% | 17,2% | 34,4% |
|  | Usually | N | 25 | 5 | 5 | 15 | 16 | 24 |
|  |  | % | 32,5% | 7,6% | 15,6% | 16,1% | 16,2% | 26,7% |
|  | Always | N | 37 | 13 | 18 | 33 | 45 | 12 |
|  |  | % | 48,1% | 19,7% | 56,3% | 35,5% | 45,5% | 13,3% |

Category 10 Rounding off the treatment

|  | | | Nationality | | | | | |
| --- | --- | --- | --- | --- | --- | --- | --- | --- |
|  |  |  | HUN | PRT | NLD | ROM | LIT | ITA |
| When your treatment in this hospital was concluded, were you informed about possible symptoms or health problems you should be aware of/watch out for? | No, not at all | N | 3 | 0 | 12 | 0 | 1 | 4 |
|  |  | % | 5,2% | 0,0% | 27,3% | 0,0% | 0,9% | 8,7% |
|  | Not really | N | 1 | 1 | 0 | 2 | 1 | 1 |
|  |  | % | 1,7% | 4,8% | 0,0% | 8,0% | 0,9% | 2,2% |
|  | More or less | N | 16 | 4 | 0 | 3 | 12 | 10 |
|  |  | % | 27,6% | 19,0% | 0,0% | 12,0% | 11,3% | 21,7% |
|  | Yes, fully | N | 38 | 16 | 32 | 20 | 92 | 31 |
|  |  | % | 65,5% | 76,2% | 72,7% | 80,0% | 86,8% | 67,4% |
| Were important people and organizations, such as your general practitioner/family doctor, homecare provider, rehabilitation centre) informed that your hospital treatment had been concluded? | No, not at all | N | 7 | 1 | 3 | 2 | 1 | 6 |
|  |  | % | 14,6% | 7,7% | 7,1% | 8,7% | 1,5% | 16,2% |
|  | Not really | N | 12 | 1 | 0 | 0 | 6 | 2 |
|  |  | % | 25,0% | 7,7% | 0,0% | 0,0% | 9,2% | 5,4% |
|  | More or less | N | 13 | 0 | 0 | 8 | 8 | 5 |
|  |  | % | 27,1% | 0,0% | 0,0% | 34,8% | 12,3% | 13,5% |
|  | Yes, fully | N | 16 | 11 | 39 | 13 | 50 | 24 |
|  |  | % | 33,3% | 84,6% | 92,9% | 56,5% | 76,9% | 64,9% |
| Were the care and support you needed at home arranged for you? | No, not at all | N | 1 | 2 | 10 | 3 | 3 | 4 |
|  |  | % | 5,3% | 22,2% | 50,0% | 13,0% | 4,4% | 17,4% |
|  | Not really | N | 3 | 2 | 0 | 2 | 5 | 2 |
|  |  | % | 15,8% | 22,2% | 0,0% | 8,7% | 7,4% | 8,7% |
|  | More or less | N | 5 | 0 | 0 | 2 | 20 | 3 |
|  |  | % | 26,3% | 0,0% | 0,0% | 8,7% | 29,4% | 13,0% |
|  | Yes, fully | N | 10 | 5 | 10 | 16 | 40 | 14 |
|  |  | % | 52,6% | 55,6% | 50,0% | 69,6% | 58,8% | 60,9% |
| Were you offered help with your questions about resuming your day-to-day activities (family, school, work) at the check-up? | Never | N | 4 | 2 | 5 | 2 | 13 | 10 |
|  |  | % | 12,5% | 16,7% | 14,3% | 9,1% | 21,7% | 30,3% |
|  | Sometimes | N | 5 | 2 | 0 | 2 | 7 | 3 |
|  |  | % | 15,6% | 16,7% | 0,0% | 9,1% | 11,7% | 9,1% |
|  | Usually | N | 14 | 1 | 0 | 5 | 10 | 11 |
|  |  | % | 43,8% | 8,3% | 0,0% | 22,7% | 16,7% | 33,3% |
|  | Always | N | 9 | 7 | 30 | 13 | 30 | 9 |
|  |  | % | 28,1% | 58,3% | 85,7% | 59,1% | 50,0% | 27,3% |
