# Supplementary material for: Piloting a generic cancer consumer quality index in six European countries
Source: BMC Cancer. 2016 Sep 2;16(1):711. doi: 10.1186/s12885-016-2752-9 (PMC5010728; doi:10.1186/s12885-016-2752-9)
Supplement: Additional file 2: — Interviewee characteristics. This file describes the characteristics of the participant to the cognitive interviews. (DOCX 14 kb) [file 12885_2016_2752_MOESM2_ESM.docx]

Additional file 2. Interviewee characteristics

| **Country** | **Participant #** | **Gender** | **Age** | **Education level** |
| --- | --- | --- | --- | --- |
| **ROM** | 1  2  3  4  5  6 | Female  Female  Female  Female  Male  Female | 45-54  45-54  25-34  18-24  45-54  55-64 | Moderate  Moderate  High  Moderate  Moderate  Moderate |
| **NLD** | 7  8  9  10  11 | Male  Male  Female  Female  Female | 55-64  65-74  45-54  45-54  18-24 | Moderate  High  High  High  High |
| **PRT** | 12  13  14 | Female  Female  Female | 55-64  55-64  45-54 | High  High  Moderate |
