# Supplementary material for: Piloting a generic cancer consumer quality index in six European countries
Source: BMC Cancer. 2016 Sep 2;16(1):711. doi: 10.1186/s12885-016-2752-9 (PMC5010728; doi:10.1186/s12885-016-2752-9)
Supplement: Additional file 3: — Problems per ECCQI-question. This file gives an overview of the problems identified per question during the cognitive interviews and through feedback on the questionnaire. (DOCX 20 kb) [file 12885_2016_2752_MOESM3_ESM.docx]

Additional file 3. Problems per European Cancer Consumer Quality Index-question/item

| **Question** | **Problem** | **Type of problem** |
| --- | --- | --- |
| Q4. For which examinations or treatment have you been to this hospital in the last 2 years? (more than one answer is possible)  ❑ Examinations, e.g. physical examination, X-ray examination, ultrasound, blood tests, CT scan, MRI scan, PET scan  ❑ Operation  ❑ Radiotherapy  ❑ Chemotherapy  ❑Hormone therapy  ❑ Immunotherapy  ❑ Aftercare  ❑ Other treatment, please state (in block capitals): | PRT: Patient 12 was missing the response category: ‘reconstruction of the breast’ | No problem, the response category: ‘operation’ could be chosen |
| Q6. When was the last time you went to this hospital for examinations, treatment or checks for cancer?  ❑ Less than 1 month ago  ❑ 1-2 months ago  ❑ 2-4 months ago  ❑ 4-8 months ago  ❑ 8-12 months ago  ❑ Over 12 months ago | ROM: Patient 1 was admitted in the hospital at that day and did not know what to answer, she missed the response category: I’m admitted today. | No problem, the response category ‘less than 1 month ago’ could be chosen |
| Q10. Was your diagnosis of cancer made at this hospital within the last 2 years?  ❑ No  ❑ Yes | NLD: Patient 2 didn’t understand the meaning of the question. | 3c. Vague |
| Q14. After diagnosis in another organisation, was it possible to start treatment as quickly as you wanted in this hospital?  ❑ No  ❑ Yes  ❑ I don’t know/I no longer remember  ❑ Not applicable | NLD: Patient 7 and 9 got their diagnoses in this hospital and didn’t know what to answer. They didn’t understand that they had to choose “Not applicable” | 2a.Conflicting or inaccurate instructions  3c. Vague |
| Q15. If you desired this, was it possible at this hospital to plan several appointments for examination and/or treatment on the same day?  ❑ Never  ❑ Sometimes  ❑ Usually  ❑ Always  ❑ I don’t know/I no longer remember  ❑ Not applicable | ROM: Patient 5 did not understand the meaning of the question.  Patient 1 (ROM), 6 (ROM) and 13 (PRT) didn’t know which kind of treatment to include. | 3c. Vague |
| Q17. Were the toilet, shower and bathroom in or near the room?  ❑Never  ❑ Sometimes  ❑ Usually  ❑ Always | ROM: Patient 2 had difficulties with this question. She never had the bathroom and the shower in the room, but always near the room. | 3c. Vague |
| Q23. Before treatment, examination or an operation began, did anyone check that you were the right person – by asking your name and date of birth, for example?  ❑ Never  ❑ Sometimes  ❑ Usually  ❑ Always  ❑ I don’t know/I no longer remember  ❑ Not applicable | ROM: For patient 1 it was confusing because the patient was asked about the name, but not about the date of birth. | 3c. Vague |
| Q32. During your treatment, were you informed about its effect ((for example whether you were responding to it)?  ❑ Never  ❑ Sometimes  ❑ Usually  ❑ Always  ❑ I don’t know/I no longer remember  ❑ Not applicable | NLD: Patient 8 had difficulties with this question. For him the example was confusing, he got information about the effect of the treatment but did not hear about the response to it. | 2a.Conflicting or inaccurate instructions |
| Q34. If you wanted, could you take part in decisions about the care and treatment you received?  ❑ Never  ❑ Sometimes  ❑ Usually  ❑ Always  ❑ Not applicable: I didn’t want to be | ROM: Patient 1 didn’t know how to answer. She thought it was a strange question, because she doesn’t thinks it is necessary to give patients the choice about the treatment.  Patient 1 and 2 didn’t think that they have the knowledge to make decisions about their treatment. | 8. Other problems |
| Q36. Were the treatment and examinations you had from different healthcare professionals well coordinated?  ❑Never  ❑ Sometimes  ❑ Usually  ❑ Always  ❑ I don’t know/I no longer remember | ROM: Patient 3, 4 and 5 had trouble with the reference period. They got the impression that the question was also about doctors in other hospitals, not only from the current hospital.  NLD: Patient 11 had difficulties with the reference period. She got the impression that the question was also about doctors in other hospitals, not only from the current hospital. | 3d. Reference periods |
| Q38. Did you always deal with the same person in this hospital – such as a doctor or nurse – when anything needed to be arranged (planning and coordinating appointments, for example)?  ❑ Never  ❑ Sometimes  ❑ Usually  ❑ Always  ❑ I don’t know/I no longer remember  ❑ Not applicable | NLD: Patient 9 and 11 thought the example of planning and coordinating appointments is confusing, because usually the secretary makes the appointments and not the doctor.  Patient 3 (ROM), 4 (ROM) and 13 (PRT) said that in the same service it was always the same person, but there were many services involved so for each service they had someone they would always speak to when there was a need to address something. | 3c. Vague |
| Q39. Were you seen by the same care providers during your investigations and treatments?  ❑ Never  ❑ Sometimes  ❑ Usually  ❑ Always  ❑ I no longer remember  ❑ Not applicable | ROM: Patient 3 didn’t know what to answer because she always received a visit from the same doctor, but from different nurses every one or two days. Patient 4 had the same problem as in question 38. | 3c. Vague |
| Q42. During aftercare, was attention paid to your pain?  ❑ Never  ❑ Sometimes  ❑ Usually  ❑ Always  ❑ I don’t know/I no longer remember  ❑ Not applicable | ROM: Patient 2, 4 and 6 had difficulties with this question, due to the translation of “aftercare”. The Romanian translation of “aftercare” means something like “outside the hospital”, this gives confusion. | 8. Other |
| Q40-45. During diagnostic phase/treatment phase/aftercare, was attention paid to your pain/fatigue?  ❑ Never  ❑ Sometimes  ❑ Usually  ❑ Always  ❑ I don’t know/I no longer remember  ❑ Not applicable | NLD: In the Dutch version ‘not applicable’ was missing. Patient 11 missed this response category because she had the diagnostic phase in another hospital.  PRT: Patient 8(NLD), 11(NLD) and 13 (PRT) had some confusion because the questions look very similar. They did not see the difference between these questions. | 7f. Missing response category  8. Other |
| Q47. Did this hospital provide you with information about help with dealing with practical problems caused by cancer and other forms of counselling on this?  ❑ Never  ❑ Sometimes  ❑ Usually  ❑ Always  ❑ I don’t know/I no longer remember  ❑ Not applicable | ROM: Patient 1 and 2 didn’t understand the meaning of the term: ‘practical problems’ | 3b. Technical term(s) |
| Q48. Did healthcare professionals inform you about patient organisations?  ❑ Never  ❑ Sometimes  ❑ Usually  ❑ Always  ❑ I don’t know/I no longer remember  ❑ Not applicable | ROM: Some patients (and even de oncologists) didn’t know what a patient organisation was.  ROM: Patient 4 said there wasn’t a need for, but didn’t choose “not applicable” | 3b. Technical term(s) |
| Q 51. Was it possible to talk to a spiritual or moral counsellor, such as a hospital chaplain or humanistic counsellor?  ❑ Never  ❑ Sometimes  ❑ Usually  ❑ Always  ❑ Not applicable in this hospital | NLD: Patient 11 missed the response category: ‘not applicable’. She said it was possible in the hospital, but she didn’t need it. | 7f. Missing response category |
| 52. Was your treatment concluded at the hospital?  ❑ No  ❑ Yes | NLD: Patient 9 and 10 didn’t knew whether or not to include “aftercare” into “treatment” | 3c. Vague |
| Q57. Were you offered help with your questions about resuming your day-to-day activities (family, school, work) at the check-up?  ❑ No  ❑ Yes  ❑ I don’t know/I no longer remember  ❑ Not applicable | **Patient 8 and 10 had difficulties with this question, because here the term “check up” is used instead of “nacontrole” like in other questions.** | **3b. Technical term(s)** |
| 61. Please indicate the length of your education (including primary education but excluding short courses) | ROM: Patient 3 didn’t know what is meant by the term: “short courses”. | 2b. Complicated instructions |
